# Extended Local-Equilibrium Thermodynamics from Chemical Jump Processes

**DOI:** 10.3390/e28101154

**Published:** 2026-10-20

**Authors:** Yannick De Decker

**Affiliations:** 1 Non-Linear Physical Chemistry Unit, Université Libre de Bruxelles (ULB), 1050 Brussels, Belgium; yannick.de.decker@ulb.be; 2 CENOLI, Université Libre de Bruxelles (ULB), 1050 Brussels, Belgium

**Keywords:** stochastic thermodynamics, chemical master equation, chemical Langevin equation, reaction networks, entropy production, Glansdorff–Prigogine criterion

## Abstract

The extended local-equilibrium approach to stochastic thermodynamics assigns thermodynamic state functions to the instantaneous fluctuating state of a system. In this work, we derive the evolution equations used in this approach from a Markov jump process for chemical reactions. We first express the changes of entropy, internal energy and free energy along individual trajectories in terms of the finite changes associated with reaction events. In the weak-noise limit, a midpoint expansion gives the Stratonovich chain rule for these state functions. We then show that the mean and variance of the entropy produced over a mesoscopic time interval obey a thermodynamic uncertainty relation. We also examine the Glansdorff–Prigogine universal evolution criterion along stochastic trajectories. Fluctuations add a contribution with no definite sign to the classical nonpositive term. Within the local Gaussian approximation, we calculate the probability that this contribution reverses the classical sign and obtain a lower bound in terms of entropy production. This bound constrains how reliably the classical sign can be observed over that interval. The results are illustrated with an open linear reaction scheme.

## 1. Introduction

Classical irreversible thermodynamics describes the evolution of a system in terms of thermodynamic state functions. Under the local-equilibrium hypothesis, these functions have the same dependence on the instantaneous state variables as at equilibrium [1,2]. Their evolution gives an expression for entropy production in terms of thermodynamic forces and fluxes. Under fixed external constraints, it also leads to the Glansdorff–Prigogine universal evolution criterion, which constrains the contribution due to changes of the forces [3,4].

For small systems, the state variables fluctuate and thermodynamic quantities must be defined in terms of stochastic processes. A connection between master equations and irreversible thermodynamics was established by Schnakenberg [5]. In the usual formulation of stochastic thermodynamics, entropy contains both a contribution associated with the mesoscopic state and a term that depends on the probability of that state [6,7,8]. This definition has led to expressions for entropy production along individual trajectories, fluctuation relations and a thermodynamic description of open chemical reaction networks [9,10,11,12,13,14,15]. Thermodynamic uncertainty relations connect entropy production with the precision of fluctuating currents [16,17,18,19]. Related results have been obtained for deterministic chemical networks [20] and reaction–diffusion systems [21]. Related stochastic studies address both the Glansdorff–Prigogine evolution criterion and stability criteria based on excess entropy production [22,23,24,25,26]. These concern different quantities: the Glansdorff–Prigogine evolution criterion constrains the force contribution during evolution, whereas excess-entropy-production criteria concern stability around a stationary state.

An alternative formulation is the extended local-equilibrium (ELE) approach [27,28,29,30]. In this approach, all thermodynamic state functions, including entropy, depend only on the instantaneous fluctuating state variables. Their evolution is obtained from the stochastic dynamics of these variables. This construction was initially introduced phenomenologically. It was later related to an Einstein–Boltzmann definition of fluctuating entropy [31].

The purpose of the present work is to derive the ELE evolution equations from the Markov jump dynamics of a reactive system. We evaluate thermodynamic state functions at the instantaneous particle numbers and express their variations as finite changes associated with individual reaction events. In the weak-noise limit, a midpoint expansion of these changes gives the Stratonovich chain rule used in the ELE approach. This relation for state functions follows from the same coarse graining in time that gives the chemical Langevin equation for the concentrations.

We then examine two consequences for entropy production. First, we obtain a relation between the mean and variance of the entropy produced over a mesoscopic time interval. This relation has the form of a known thermodynamic uncertainty relation [18]. Here, we derive it for the Einstein–Boltzmann entropy from the forward and reverse reaction rates. Second, we consider the contribution to the variation of entropy production that is due to changes of the thermodynamic forces. The Glansdorff–Prigogine criterion requires this contribution to be nonpositive in the macroscopic description. Along a stochastic trajectory, an additional contribution with no definite sign appears. We calculate its variance and, within the local Gaussian approximation, the probability that the classical sign is reversed. We also relate this probability to the entropy produced over the same interval.

The results are illustrated with an open linear reaction scheme. We compare Gillespie simulations of the jump process with the weak-noise description and examine the fluctuations of entropy production, the uncertainty relation and the probability of a reversal of the Glansdorff–Prigogine sign. The same example allows us to compare the fluctuations of the time-averaged entropy production with earlier ELE results around a nonequilibrium stationary state.

The paper is organized as follows. Section 2 introduces the stochastic reaction dynamics and its weak-noise limit. In Section 3, we define thermodynamic state functions along trajectories and derive their ELE evolution equations. Section 4 is devoted to entropy-production fluctuations and the Glansdorff–Prigogine criterion. Section 5 presents the linear reaction scheme and the numerical comparisons. Conclusions are given in Section 6.

## 2. Mesoscopic Reactive Dynamics

We consider a spatially uniform reactive system at constant volume and temperature, in the absence of bulk motion and external forces. The system can be maintained out of equilibrium thanks to exchanges with external reservoirs where concentrations are maintained constant (chemostats).

The stochastic variables are the particle numbers of the internal species, $N=(N_{1},\ldots ,N_{n})$. Chemostatted species are excluded from this state vector. Their imposed concentrations enter the transition probabilities as fixed parameters. The particle numbers $N_{i}$ change through the chemical processes occurring in the system. One occurrence of process *r* changes the number of particles of species *i* according to

(1) $$N_{i}⟶N_{i}+\nu_{ir},$$

where $\nu_{ir}$ is the stoichiometric coefficient of species *i* in process *r*. The stoichiometric coefficients form the vector $\nu_{r}=\left(\nu_{1r},\ldots ,\nu_{nr}\right)$. Each elementary process is taken to be reversible, with $\nu_{i,-r}=-\nu_{ir}$ for the reverse process −r.

With this convention, the chemical master equation for the probability P(N,t) of having the state vector N at time *t* is

(2) $$\frac{dP(N,t)}{dt}=\sum_{r=\pm 1,\ldots ,\pm R}\left[W_{r}\left(N-\nu_{r}\right)\;P\left(N-\nu_{r},t\right)-W_{r}\left(N\right)\;P\left(N,t\right)\right],$$

where $W_{r}\left(N\right)$ are transition probabilities per unit time. The same dynamics can be expressed in terms of counting processes. Let $N_{r}\left(t\right)$ be the number of occurrences of process *r* up to time *t*, and let $dN_{r}\left(t\right)=N_{r}\left(t+dt\right)-N_{r}\left(t\right)$. For an infinitesimal time increment dt,

(3) $$\mathrm{Prob}\left\{dN_{r}\left(t\right)=1|N\left(t^{-}\right)=N\right\}=W_{r}\left(N\right)\;dt+o\left(dt\right),$$

where $N(t^{-})$ is the state immediately before *t*; the probability of two or more occurrences is o(dt). For each reversible pair of processes, with r=1,…,R denoting one chosen orientation, we now introduce the degree of advancement,

(4) $$d\xi_{r}\left(t\right)=dN_{r}\left(t\right)-dN_{-r}\left(t\right),$$

which counts the net number of forward events. The jump equation for the particle numbers can then be written as

(5) $$dN_{i}\left(t\right)=\sum_{r=1}^{R}\nu_{ir}\;d\xi_{r}\left(t\right).$$

Finally, introducing the volume *V*, the concentrations $c_{i}=N_{i}/V$ obey

(6) $$dc_{i}\left(t\right)=\frac{1}{V}\sum_{r=1}^{R}\nu_{ir}\;d\xi_{r}\left(t\right).$$

From now on, we show the time argument only for stochastic processes such as N(t), $\xi_{r}\left(t\right)$ and c(t). The rates depend on time through the instantaneous state.

### Weak-Noise Limit

We now consider the large-*V*, or weak-noise, limit. The transition probabilities per unit time are assumed to admit the size expansion

(7) $$W_{r}\left(N\right)=V\;w_{r}\left(c\right)+w_{r}^{\left(1\right)}\left(c\right)+O\left(V^{-1}\right),$$

where $w_{r}\left(c\right)$ is the macroscopic rate per unit volume. The first correction $w_{r}^{\left(1\right)}\left(c\right)$ accounts, in particular, for finite-number combinatorial corrections in the transition probabilities of nonlinear processes. For each reversible pair of elementary processes, we also define the net rate and the rate sum by

(8) $$v_{r}\left(c\right)=w_{r}\left(c\right)-w_{-r}\left(c\right),\;Q_{r}\left(c\right)=w_{r}\left(c\right)+w_{-r}\left(c\right),$$

and the first correction to the net rate by

(9) $$v_{r}^{\left(1\right)}\left(c\right)=w_{r}^{\left(1\right)}\left(c\right)-w_{-r}^{\left(1\right)}\left(c\right).$$

The weak-noise approximation involves a coarse-graining in time in addition to the large-*V* expansion. Following Gillespie, we consider a “mesoscopic” time interval Δt that is short enough for the transition probabilities to remain approximately constant, but long enough for the relevant elementary processes to occur many times,

(10) $$W_{r}\left(N\right)\;\Delta t\gg 1.$$

The numbers of occurrences during this interval can then be approximated by Gaussian variables with the same mean and variance [32]. For the degree of advancement $\Delta \xi_{r}$ of each reaction, one consequently has (to the orders retained here)

(11) $$\Delta \xi_{r}=\left[V\;v_{r}\left(c\right)+v_{r}^{\left(1\right)}\left(c\right)\right]\Delta t+\sqrt{V\;Q_{r}\left(c\right)\;\Delta t}\;Z_{r},$$

where $Z_{r}$ are independent standard Gaussian variables. Assuming Δt to be small compared to the macroscopic time scale, this coarse-grained dynamics can be represented by the stochastic differential equation

(12) $$d\xi_{r}\left(t\right)=\left[V\;v_{r}\left(c\right)+v_{r}^{\left(1\right)}\left(c\right)\right]dt+\sqrt{V\;Q_{r}\left(c\right)}\;dZ_{r}\left(t\right),$$

where the $Z_{r}\left(t\right)$ are independent Wiener processes. Since the rates are evaluated at the beginning of the interval, this equation has an Itô interpretation. A Stratonovich representation would change the drift of $\xi_{r}$ at order 1, or that of $\xi_{r}/V$ at order $V^{-1}$. In Section 3, we consider how the changes of thermodynamic state functions follow from this dynamics.

Using Equation (6), the concentrations obey

(13) $$dc_{i}\left(t\right)=\sum_{r=1}^{R}\nu_{ir}\left[v_{r}\left(c\right)+\frac{1}{V}\;v_{r}^{\left(1\right)}\left(c\right)\right]dt+\frac{1}{\sqrt{V}}\sum_{r=1}^{R}\nu_{ir}\;\sqrt{Q_{r}\left(c\right)}\;dZ_{r}\left(t\right).$$

Equation (13) is the chemical Langevin equation, with the first finite-size correction to the deterministic drift retained [32,33,34,35,36,37,38,39]. Note that a first correction to the rate sum $Q_{r}$ would modify the concentration noise only at order $V^{-3/2}$ and is therefore not required at the present order.

For later use, we introduce the fluctuating contribution to the reaction rate through

(14) $${\tilde{v}}_{r}\left(t\right)\;dt=\frac{1}{\sqrt{V}}\;\sqrt{Q_{r}\left(c\right)}\;dZ_{r}\left(t\right).$$

Equation (13) may then be written as

(15) $$dc_{i}\left(t\right)=\sum_{r=1}^{R}\nu_{ir}\left[v_{r}\left(c\right)+\frac{1}{V}\;v_{r}^{\left(1\right)}\left(c\right)+{\tilde{v}}_{r}\left(t\right)\right]dt.$$

Here, $v_{r}+v_{r}^{\left(1\right)}/V$ is the systematic part of the reaction rate and ${\tilde{v}}_{r}$ is its fluctuating part. This separation will also appear in the entropy production.

## 3. Thermodynamic Quantities Along Trajectories

We now evaluate the thermodynamic state functions at the instantaneous mesoscopic state. Consider an arbitrary state function F(N). For any elementary process *r*, we define its finite change by

(16) $$\Delta_{r}F\left(N\right)=F\left(N+\nu_{r}\right)-F\left(N\right).$$

Along a stochastic trajectory, the corresponding increment is therefore

(17) $$dF\left(t\right)=\sum_{r=\pm 1,\ldots ,\pm R}\Delta_{r}F\left(N\right)\;dN_{r}\left(t\right).$$

Equation (17) is exact for the jump process. In such expressions, N denotes the state immediately preceding the elementary event.

### 3.1. Einstein–Boltzmann Entropy of a Mesoscopic State

Consider an equilibrium reference at fixed temperature, volume and chemical potentials. For a given N, let Z(N) be the canonical partition function constrained by these particle numbers. The grand-canonical probability is

(18) $$P\left(N,\mathrm{eq}\right)=Z\left(N\right)\;\exp\left\{\beta \left[\Omega_{\mathrm{eq}}+\sum_{i}\mu_{i}\;N_{i}\right]\right\},$$

where $\beta =1/(k_{B}\;T)$, $\Omega_{\mathrm{eq}}$ is the equilibrium grand potential and $\mu_{i}$ are the chemical potentials. Einstein’s fluctuation formula expresses the same probability in terms of the thermodynamic potential:

(19) $$P\left(N,\mathrm{eq}\right)\propto \exp\left[-\beta \;\Delta \Omega (N)\right],$$

where

(20) $$\Delta \Omega \left(N\right)=\Omega \left(N\right)-\Omega_{\mathrm{eq}}.$$

Comparing Equations (18) and (19) gives

(21) $$\Omega \left(N\right)=A-k_{B}\;T\;\ln Z\left(N\right)-\sum_{i}\mu_{i}\;N_{i},$$

where *A* is independent of N. Since the grand potential is also given by

(22) $$\Omega \left(N\right)=F\left(N\right)-\sum_{i}\mu_{i}\;N_{i},$$

one obtains

(23) $$F\left(N\right)=A-k_{B}\;T\;\ln Z\left(N\right).$$

If U(N) is the corresponding statewise internal energy, the entropy assigned to the mesoscopic state is therefore

(24) $$S\left(N\right)=\frac{U(N)-F(N)}{T}=\frac{U(N)}{T}+k_{B}\;\ln Z\left(N\right)-\frac{A}{T}.$$

Equation (24) defines the entropy used in this work [31]. It is the thermodynamic entropy associated with the microscopic states compatible with the composition N. It depends on N, but not explicitly on its probability distribution. Entropy fluctuates because N fluctuates. Away from equilibrium, we retain this dependence on the particle numbers, as in the local-equilibrium hypothesis. The equilibrium reference used to define the state functions does not require the driven system to have an equilibrium stationary state.

### 3.2. Entropy Balance Along a Trajectory

The state functions U(N), F(N) and S(N) all obey Equation (17). We now consider entropy and separate its variation into entropy exchange and entropy production. Here S(N) is the thermodynamic state entropy defined above; it does not include the self-information term $-k_{B}\;\ln P\left(N\left(t\right),t\right)$ that enters the trajectory entropy of standard stochastic thermodynamics.

The entropy change of the system along a trajectory is given by

(25) $$dS\left(t\right)=\sum_{r=\pm 1,\ldots ,\pm R}\Delta_{r}S\left(N\right)\;dN_{r}\left(t\right).$$

From this point, we denote chemical processes by ρ and chemostatted species by α. The coefficient $\nu_{\alpha \rho }$ is the stoichiometric coefficient of species α in process ρ. The exchanges that maintain the chemostatted concentrations do not appear as separate jumps of N, since these species are excluded from the state vector. Their contribution to the entropy balance is expressed through their fixed chemical potentials. The entropy variation can be written as

(26) $$dS=d_{e}S+d_{i}S,$$

where

(27) $$d_{e}S\left(t\right)=\sum_{\rho }\frac{1}{T}\left[\Delta_{\rho }U\left(N\right)+\sum_{\alpha \in \mathrm{ch}}\nu_{\alpha \rho }\;\mu_{\alpha }\right]dN_{\rho }\left(t\right).$$

The first term accounts for the energy exchanged to maintain the temperature. The second accounts for the matter exchanges that maintain the chemical potentials of the chemostatted species.

The entropy production is, consequently,

(28) $$\begin{array}{cc} d_{i}S\left(t\right)\; & =\sum_{\rho }\left[\Delta_{\rho }S\left(N\right)-\frac{\Delta_{\rho }U\left(N\right)}{T}-\sum_{\alpha \in \mathrm{ch}}\frac{\nu_{\alpha \rho }\;\mu_{\alpha }}{T}\right]dN_{\rho }\left(t\right)\\ \; & =-\sum_{\rho }\frac{1}{T}\left[\Delta_{\rho }F\left(N\right)+\sum_{\alpha \in \mathrm{ch}}\nu_{\alpha \rho }\;\mu_{\alpha }\right]dN_{\rho }\left(t\right), \end{array}$$

where F=U−TS. The chemical potentials of the chemostatted species appear separately because these species are excluded from N. If they were retained, as in Ref. [31], their contribution would be included in the free-energy change of the enlarged system. We introduce the mesoscopic affinity of process ρ as

(29) $${\tilde{A}}_{\rho }\left(N\right)=-\left[\Delta_{\rho }F\left(N\right)+\sum_{\alpha \in \mathrm{ch}}\nu_{\alpha \rho }\;\mu_{\alpha }\right].$$

The same combination of a free-energy change and chemostat chemical potentials appears in recent stochastic-thermodynamic treatments of open reaction networks [40]. The entropy production then takes the simple form

(30) $$d_{i}S\left(t\right)=\sum_{\rho }\frac{{\tilde{A}}_{\rho }\left(N\right)}{T}\;dN_{\rho }\left(t\right).$$

The mesoscopic affinity can also be related to the transition probabilities per unit time. At equilibrium, detailed balance gives

(31) $$W_{\rho }\left(N\right)\;P\left(N,\mathrm{eq}\right)=W_{-\rho }\left(N+\nu_{\rho }\right)\;P\left(N+\nu_{\rho },\mathrm{eq}\right).$$

Using the equilibrium distribution introduced above and the equilibrium condition

(32) $$\sum_{i}\nu_{i\rho }\;\mu_{i}+\sum_{\alpha \in \mathrm{ch}}\nu_{\alpha \rho }\;\mu_{\alpha }=0,$$

one obtains

(33) $$\frac{{\tilde{A}}_{\rho }\left(N\right)}{T}=k_{B}\;\ln\frac{W_{\rho }\left(N\right)}{W_{-\rho }\left(N+\nu_{\rho }\right)}.$$

For the driven system, we impose Equation (33) as the local detailed-balance condition for each elementary reaction at the maintained reservoir chemical potentials. Equilibrium detailed balance motivates this relation, but does not by itself determine how arbitrary rates change away from equilibrium.

### 3.3. Weak-Noise Limit and the Extended Local Equilibrium Framework

We now consider the weak-noise limit of the state-function relation (17). For an extensive thermodynamic state function F, we introduce the corresponding density

(34) $$\varphi =\frac{F}{V}.$$

Over the mesoscopic interval Δt introduced in Section 2, the concentration increment is

(35) $$\Delta c_{i}=\sum_{r}\nu_{ir}\left[v_{r}\left(c\right)+\frac{1}{V}\;v_{r}^{\left(1\right)}\left(c\right)\right]\Delta t+\frac{1}{\sqrt{V}}\sum_{r}\nu_{ir}\;\sqrt{Q_{r}\left(c\right)}\;\Delta Z_{r}.$$

Since φ is a state function of c, its change during the same time interval depends only on the concentrations at the beginning and at the end of the interval:

(36) Δφ=φ(c+Δc)−φ(c).

Expanding to second order gives

(37) $$\begin{array}{cc} \Delta \varphi \; & =\sum_{i}{\left(\frac{\partial \varphi }{\partial c_{i}}\right)}_{c}\;\Delta c_{i}+\frac{1}{2}\sum_{ij}{\left(\frac{\partial^{2}\varphi }{\partial c_{i}\;\partial c_{j}}\right)}_{c}\;\Delta c_{i}\;\Delta c_{j}+\cdots \\ \; & =\sum_{i}\left[{\left(\frac{\partial \varphi }{\partial c_{i}}\right)}_{c}+\frac{1}{2}\sum_{j}{\left(\frac{\partial^{2}\varphi }{\partial c_{i}\;\partial c_{j}}\right)}_{c}\;\Delta c_{j}\right]\Delta c_{i}+\cdots . \end{array}$$

Equation (37) can also be written as

(38) $$\Delta \varphi =\sum_{i}{\left(\frac{\partial \varphi }{\partial c_{i}}\right)}_{c+\Delta c2}\;\Delta c_{i}+\cdots .$$

The derivative is thus evaluated, to the retained order, at the midpoint between the concentrations at the beginning and at the end of the coarse-grained time interval. In the continuous-time limit, this midpoint prescription corresponds to the Stratonovich interpretation, and one obtains

(39) $$d\varphi =\sum_{i}\left(\frac{\partial \varphi }{\partial c_{i}}\right)\circ dc_{i}.$$

Equation (39) is the relation used for state functions in the ELE framework. The Stratonovich product appears because the finite change of a state function gives a midpoint derivative. This is compatible with the Itô equation for the concentrations: the Stratonovich product in Equation (39) is taken with respect to $dc_{i}$.

The quadratic term contributes to the drift at order $V^{-1}$. The finite-size corrections to the thermodynamic densities and the kinetic correction $v_{r}^{\left(1\right)}/V$ occur at the same order. In the ELE equations below, we retain the macroscopic drift and the leading fluctuations of order $V^{-1/2}$. Drift corrections of order $V^{-1}$ are beyond this approximation.

For the internal energy density, Equation (39) gives

(40) $$du=\sum_{i}{\left(\frac{\partial u}{\partial c_{i}}\right)}_{T,V,c_{j\neq i}}\circ dc_{i},$$

The Helmholtz free energy density obeys

(41) $$df=\sum_{i}{\left(\frac{\partial f}{\partial c_{i}}\right)}_{T,V,c_{j\neq i}}\circ dc_{i},$$

and the entropy density obeys

(42) $$ds=\sum_{i}{\left(\frac{\partial s}{\partial c_{i}}\right)}_{T,V,c_{j\neq i}}\circ dc_{i},$$

The internal concentrations change through the chemical processes, so that

(43) $$dc_{i}=\frac{1}{V}\sum_{\rho }\nu_{i\rho }\;d\xi_{\rho },$$

where the sum is over one chosen orientation of each reversible pair, as in Equation (6). Equation (42) consequently gives

(44) $$ds=\sum_{\rho }\left[\sum_{i}\nu_{i\rho }\;{\left(\frac{\partial s}{\partial c_{i}}\right)}_{T,V,c_{j\neq i}}\right]\circ \frac{d\xi_{\rho }}{V}.$$

The entropy exchange associated with the same reaction increments is

(45) $$d_{e}s=\frac{1}{T}\sum_{\rho }\left[\sum_{i}\nu_{i\rho }\;{\left(\frac{\partial u}{\partial c_{i}}\right)}_{T,V,c_{j\neq i}}+\sum_{\alpha \in \mathrm{ch}}\nu_{\alpha \rho }\;\mu_{\alpha }\right]\circ \frac{d\xi_{\rho }}{V}.$$

The two terms have the same interpretation as in the jump description: they account for energy and matter exchanges with the reservoirs.

Subtracting Equation (45) from Equation (44) gives the entropy production,

(46) $$\begin{array}{cc} d_{i}s & =\sum_{\rho }\left[\sum_{i}\nu_{i\rho }\;{\left(\frac{\partial s}{\partial c_{i}}\right)}_{T,V,c_{j\neq i}}-\frac{1}{T}\sum_{i}\nu_{i\rho }\;{\left(\frac{\partial u}{\partial c_{i}}\right)}_{T,V,c_{j\neq i}}-\frac{1}{T}\sum_{\alpha \in \mathrm{ch}}\nu_{\alpha \rho }\;\mu_{\alpha }\right]\circ \frac{d\xi_{\rho }}{V}. \end{array}$$

The first two terms combine into minus the chemical potential of species *i* since, by definition

(47) $$\mu_{i}\left(c\right)={\left(\frac{\partial f}{\partial c_{i}}\right)}_{T,V,c_{j\neq i}}={\left(\frac{\partial u}{\partial c_{i}}\right)}_{T,V,c_{j\neq i}}-T\;{\left(\frac{\partial s}{\partial c_{i}}\right)}_{T,V,c_{j\neq i}},$$

and Equation (46) becomes

(48) $$d_{i}s=\sum_{\rho }\frac{A_{\rho }}{T}\circ \frac{d\xi_{\rho }}{V},$$

where

(49) $$A_{\rho }=-\sum_{i}\nu_{i\rho }\;\mu_{i}\left(c\right)-\sum_{\alpha \in \mathrm{ch}}\nu_{\alpha \rho }\;\mu_{\alpha }$$

is the usual thermodynamic affinity. Equation (48) is the stochastic extension of the local-equilibrium expression for entropy production used in the ELE framework [28,29].

To relate this affinity to the mesoscopic affinity, consider one occurrence of process ρ. The concentrations change by $\nu_{\rho }/V$. Using f=F/V, the midpoint expansion gives

(50) $$\Delta_{\rho }F\left(N\right)=\sum_{i}\nu_{i\rho }\;\mu_{i}\left(c+\frac{\nu_{\rho }}{2\;V}\right)+O\left(V^{-1}\right),$$

where the remainder includes the finite-size corrections to the thermodynamic density discussed above. The mesoscopic affinity (29) consequently becomes

(51) $$\begin{array}{cc} {\tilde{A}}_{\rho }\left(N\right)\; & =-\sum_{i}\nu_{i\rho }\;\mu_{i}\left(c+\frac{\nu_{\rho }}{2\;V}\right)-\sum_{\alpha \in \mathrm{ch}}\nu_{\alpha \rho }\;\mu_{\alpha }+O\left(V^{-1}\right)\\ \; & =A_{\rho }\left(c+\frac{\nu_{\rho }}{2\;V}\right)+O\left(V^{-1}\right)\\ \; & =A_{\rho }\left(c\right)+O\left(V^{-1}\right). \end{array}$$

The mesoscopic affinity thus reduces to the usual thermodynamic affinity in the weak-noise limit. The difference between them is of order $V^{-1}$.

We now use the separation of the reaction rates introduced in Section 2. At the order retained in the ELE approximation, Equation (12) reads

(52) $$\frac{1}{V}\;d\xi_{\rho }=\left[v_{\rho }\left(c\right)+{\tilde{v}}_{\rho }\left(t\right)\right]dt.$$

Substitution into Equation (48) gives

(53) $$d_{i}s=\sigma_{I}\;dt+\sigma_{\mathrm{II}}\;dt,$$

where

(54) $$\sigma_{I}=\sum_{\rho }\frac{A_{\rho }\left(c\right)}{T}\;v_{\rho }\left(c\right)$$

is called the systematic entropy-production rate, while

(55) $$\sigma_{\mathrm{II}}=\sum_{\rho }\frac{A_{\rho }\left(c\right)}{T}\circ {\tilde{v}}_{\rho }\left(t\right)$$

is the contribution from the fluctuating reaction rates. The term $\sigma_{I}$ also fluctuates, through its dependence on the composition; the distinction refers to the two parts of the reaction rates. This decomposition was introduced phenomenologically in the ELE approach [28,29]. Here, it follows from the weak-noise limit of the jump process. In the macroscopic limit, $\sigma_{\mathrm{II}}$ vanishes and $\sigma_{I}$ reduces to the classical entropy-production rate.

We now examine the properties of this entropy production.

## 4. Systematic Entropy Production and Universal Evolution

We first establish a relation between the mean and variance of entropy-production increments. We then consider how fluctuations affect the contribution to the variation of entropy production that enters the Glansdorff–Prigogine criterion.

### 4.1. Systematic Entropy Production and Its Fluctuations

The entropy-production increment obtained in Section 3 contains the systematic and fluctuating contributions defined in Equations (54) and (55). We first show that $\sigma_{I}$ is non-negative.

In the weak-noise limit, Equations (33) and (51) give

(56) $$\frac{A_{\rho }\left(c\right)}{T}=k_{B}\;\ln\frac{w_{\rho }\left(c\right)}{w_{-\rho }\left(c\right)}.$$

The systematic entropy-production rate can therefore be written as

(57) $$\sigma_{I}\left(c\right)=k_{B}\;\sum_{\rho }\left[w_{\rho }\left(c\right)-w_{-\rho }\left(c\right)\right]\ln\frac{w_{\rho }\left(c\right)}{w_{-\rho }\left(c\right)}\geq 0.$$

Each term in the sum is non-negative. Hence, although $\sigma_{I}$ fluctuates through its dependence on the instantaneous composition, it remains non-negative at every composition. It vanishes only when the forward and reverse rates are equal for every reversible reaction pair.

The increment $\Delta_{i}s$, which includes both contributions, need not be positive along each realization. We evaluate its moments over the mesoscopic interval Δt, for a given composition c at the beginning of the interval. At the retained order, Equation (55) gives

(58) $$\Delta_{i}s=\sigma_{I}\left(c\right)\;\Delta t+\frac{1}{\sqrt{V}}\sum_{\rho }\frac{A_{\rho }\left(c\right)}{T}\;\sqrt{Q_{\rho }\left(c\right)}\;\Delta Z_{\rho }.$$

With the coefficients evaluated at c, the second term has zero mean but no definite sign. The average over realizations starting at this composition is therefore

(59) $$\langle \Delta_{i}s\rangle =\sigma_{I}\left(c\right)\;\Delta t$$

and

(60) $$\frac{\mathrm{Var}(\Delta_{i}s)}{\Delta t}=\frac{1}{V}\sum_{\rho }Q_{\rho }\left(c\right){\left[\frac{A_{\rho }\left(c\right)}{T}\right]}^{2}.$$

The mean and variance depend on the same affinities, but involve different combinations of reaction rates. The mean contains the net rates, while the variance contains the sums $Q_{\rho }=w_{\rho }+w_{-\rho }$. Both forward and reverse events contribute to the fluctuations, even when their mean effects partly cancel.

Local detailed balance makes it possible to relate these two quantities directly. From Equation (56),

(61) $$v_{\rho }\left(c\right)=Q_{\rho }\left(c\right)\;\tanh\;\left[\frac{A_{\rho }\left(c\right)}{2\;k_{B}\;T}\right].$$

Using |tanhx|≤|x|, one obtains for every reaction pair

(62) $$Q_{\rho }\left(c\right){\left[\frac{A_{\rho }\left(c\right)}{T}\right]}^{2}\geq 2\;k_{B}\;\frac{A_{\rho }\left(c\right)}{T}\;v_{\rho }\left(c\right).$$

Summing over the reaction pairs and using Equations (54) and (60) gives

(63) $$\mathrm{Var}\left(\Delta_{i}s\right)\geq \frac{2\;k_{B}}{V}\;\langle \Delta_{i}s\rangle .$$

Equality is approached close to equilibrium, where $\tanh x=x+O(x^{3})$.

For the entropy produced in the whole volume,

(64) $$\Delta_{i}S=V\;\Delta_{i}s,$$

Equation (63) becomes

(65) $$\frac{\mathrm{Var}(\Delta_{i}S)}{\langle \Delta_{i}S\rangle }\geq 2\;k_{B}.$$

Equation (65) has the form of a thermodynamic uncertainty relation. The first such relations connected the precision of stationary currents, measured over long times, to entropy production [16,17]. Extensions to finite observation times were subsequently obtained [19]. The entropy-production bound in Equation (65) is already known in this context [18]. Here, we obtain it for the Einstein–Boltzmann entropy production over the mesoscopic interval used in the weak-noise approximation. The result is local in composition and does not require a stationary state.

The inequality means that, for a given mean entropy production, the fluctuations cannot be arbitrarily small. In comparing this result with standard stochastic thermodynamics, one must also distinguish the definitions of entropy. For the same entropy exchange, the usual trajectory entropy production contains the additional term $-k_{B}\;\Delta \ln P\left(N\left(t\right),t\right)$ [6,7,8]. The bound derived here concerns the ELE increment over Δt; it does not establish a bound at arbitrary times for that other definition.

### 4.2. The Glansdorff–Prigogine Universal Evolution Criterion at Finite Volume

The Glansdorff–Prigogine (GP) universal evolution criterion applies to nonequilibrium systems evolving under fixed external constraints [2,3,4]. For a deterministic chemical reaction network, the entropy-production rate is

(66) $$\sigma =\sum_{\rho }\frac{A_{\rho }}{T}\;v_{\rho }.$$

Its variation can be separated into contributions associated with changes of the thermodynamic forces and of the reaction rates. Following the usual notation, $d_{X}\sigma$ denotes the contribution due to changes of the forces $A_{\rho }/T$, with the reaction rates kept as coefficients. The universal evolution criterion concerns this contribution and states that

(67) $$\frac{d_{X}\sigma }{dt}=\sum_{\rho }v_{\rho }\;\frac{d}{dt}\left(\frac{A_{\rho }}{T}\right)\leq 0.$$

This does not imply that the entropy-production rate itself decreases, since the contribution associated with changes of the reaction rates has no definite sign.

Previous stochastic studies have considered fluctuations around nonequilibrium states and the general evolution criterion [22,25]. Other work concerns the stability criterion expressed through excess entropy production [23,24,26]. We consider here the force contribution in Equation (67) along individual trajectories and calculate the probability that its increment is positive.

The part of the variation of $\sigma_{I}$ due to changes of the affinities is

(68) $$d_{X}\sigma_{I}=\sum_{\rho }v_{\rho }\left(c\right)\circ d\;\left(\frac{A_{\rho }\left(c\right)}{T}\right).$$

Since the chemical potentials of the chemostatted species are fixed, Equation (49) gives

(69) $$d\;\left(\frac{A_{\rho }}{T}\right)=-\frac{1}{T}\sum_{ij}\nu_{i\rho }\;\left(\frac{\partial \mu_{i}}{\partial c_{j}}\right)\circ dc_{j}.$$

Introducing the deterministic rate of change in the concentrations,

(70) $${\dot{c}}_{i}=\sum_{\rho }\nu_{i\rho }\;v_{\rho },$$

Equation (68) becomes

(71) $$d_{X}\sigma_{I}=-\frac{1}{T}\sum_{ij}{\dot{c}}_{i}\;\left(\frac{\partial \mu_{i}}{\partial c_{j}}\right)\circ dc_{j}.$$

For the deterministic evolution, this is minus the quadratic form of $\dot{c}$ associated with the matrix $T^{-1}\partial \mu_{i}/\partial c_{j}$. Its nonpositive sign requires the Helmholtz free-energy density to be convex on the accessible composition variations. We assume this thermodynamic stability condition throughout; symmetry of the matrix alone would not suffice. For an ideal mixture at positive concentrations, $\partial \mu_{i}/\partial c_{j}=k_{B}\;T\;\delta_{ij}/c_{i}$, so the condition is satisfied.

To the order retained here, the chemical Langevin equation may be written in Stratonovich form as

(72) $$dc_{j}={\dot{c}}_{j}\;dt+\frac{1}{\sqrt{V}}\sum_{\rho }\nu_{j\rho }\;\sqrt{Q_{\rho }}\circ dZ_{\rho }+O\left(V^{-1}\right)\;dt.$$

The convention-dependent drift correction is of order $V^{-1}$ and is included in the remainder. Using the symmetry of the matrix $\partial \mu_{i}/\partial c_{j}$, substitution into Equation (71) gives

(73) $$d_{X}\sigma_{I}=\sum_{\rho }v_{\rho }\;\frac{d}{dt}\left(\frac{A_{\rho }}{T}\right)\;dt+\frac{1}{\sqrt{V}}\sum_{\rho }\sqrt{Q_{\rho }}\;\frac{d}{dt}\left(\frac{A_{\rho }}{T}\right)\circ dZ_{\rho }+O\left(V^{-1}\right)\;dt.$$

Here, the time derivatives of the affinities are evaluated using the deterministic concentration rates ${\dot{c}}_{i}$. They are not time derivatives of the fluctuating trajectories.

The first term in Equation (73) is the classical Glansdorff–Prigogine contribution and is nonpositive. The second is of order $V^{-1/2}$ and has no definite sign. Its meaning can be seen from Equation (71): the fluctuating displacement is combined with the deterministic rate of change of the composition, with weights given by $T^{-1}\partial \mu_{i}/\partial c_{j}$. A displacement along the deterministic direction makes the contribution more negative. A displacement in the opposite direction can reverse its sign.

For a given initial composition, the increment over Δt is Gaussian in the same local approximation as above. Its mean is

(74) $$\langle \Delta_{X}\sigma_{I}\rangle =\left[\sum_{\rho }v_{\rho }\;\frac{d}{dt}\left(\frac{A_{\rho }}{T}\right)\right]\Delta t\leq 0,$$

while its variance is

(75) $$\frac{\mathrm{Var}\left(\Delta_{X}\sigma_{I}\right)}{\Delta t}=\frac{1}{V}\sum_{\rho }Q_{\rho }{\left[\frac{d}{dt}\left(\frac{A_{\rho }}{T}\right)\right]}^{2}.$$

These moments give the probability of a positive increment in the local Gaussian approximation:

(76) $$\mathrm{Prob}\left(\Delta_{X}\sigma_{I}>0\right)=\frac{1}{2}\;\mathrm{erfc}\left[\frac{\left|\langle \Delta_{X}\sigma_{I}\rangle \right|}{\sqrt{2\;\mathrm{Var}\left(\Delta_{X}\sigma_{I}\right)}}\right].$$

This probability can itself be related to the entropy production. A weighted Cauchy–Schwarz inequality gives

(77) $${\left[\sum_{\rho }v_{\rho }\;\frac{d}{dt}\left(\frac{A_{\rho }}{T}\right)\right]}^{2}\leq \left(\sum_{\rho }\frac{v_{\rho }^{2}}{Q_{\rho }}\right)\left\{\sum_{\rho }Q_{\rho }{\left[\frac{d}{dt}\left(\frac{A_{\rho }}{T}\right)\right]}^{2}\right\}.$$

Furthermore, Equation (61) and $2\tanh^{2}\left(x/2\right)\leq x\tanh\left(x/2\right)$ imply

(78) $$\sum_{\rho }\frac{v_{\rho }^{2}}{Q_{\rho }}\leq \frac{\sigma_{I}}{2\;k_{B}}.$$

Using Equations (74) and (75), we therefore obtain

(79) $$\frac{{\left|\langle \Delta_{X}\sigma_{I}\rangle \right|}^{2}}{\mathrm{Var}\left(\Delta_{X}\sigma_{I}\right)}\leq \frac{\langle \Delta_{i}S\rangle }{2\;k_{B}},$$

where $\langle \Delta_{i}S\rangle =V\;\sigma_{I}\;\Delta t$ is the mean total entropy produced over the same interval. Since the complementary error function is monotonically decreasing, Equation (76) consequently yields

(80) $$\mathrm{Prob}\left(\Delta_{X}\sigma_{I}>0\right)\geq \frac{1}{2}\;\mathrm{erfc}\left[\sqrt{\frac{\langle \Delta_{i}S\rangle }{4\;k_{B}}}\right].$$

At the retained order, the mean GP contribution has the classical sign, but individual increments can have the opposite sign. Equation (79) has the form of a thermodynamic uncertainty relation: as in uncertainty relations for fluctuating currents [16,17,18,19], entropy production bounds a squared mean-to-variance ratio. Here the fluctuating quantity is the force contribution entering the Glansdorff–Prigogine criterion. Within the local Gaussian approximation, this gives the lower bound (80) on the reversal probability.

The bound gives a physical interpretation of the classical sign at finite volume. Within the local Gaussian approximation and where the variance is nonzero, a small amount of entropy production over the observation interval prevents the probability of a positive GP increment from becoming arbitrarily small. Increasing the entropy production lowers this restriction, but does not by itself guarantee a small reversal probability. The bound therefore gives a necessary dissipation requirement for observing the classical sign with a prescribed reliability. These reversals concern the force contribution to the variation of entropy production; they imply neither a violation of the second law nor a loss of dynamical stability.

At a deterministic stationary state,

(81) $${\dot{c}}_{i}=0$$

for every internal species. The mean and variance in Equations (74) and (75) then both vanish at the retained order, even if reaction currents and entropy production remain nonzero. Equation (76) therefore does not determine the probability at the stationary composition. In the example below, we test the GP result away from that composition.

## 5. Example

We illustrate the results with the linear reaction scheme

(82) A⇌X⇌B,

where *A* and *B* are chemostatted. The jump process can be simulated with the Gillespie algorithm and compared with the ELE description. This simple scheme retains an essential feature of open reaction networks: a stationary internal concentration can coexist with a nonzero current from *A* to *B* and a positive entropy-production rate. The separate degrees of advancement record this conversion even when the mean population of *X* is constant. Retaining the reaction channels therefore matters for the thermodynamic description.

We first consider entropy-production fluctuations and the Glansdorff–Prigogine contribution over a mesoscopic time interval. We then examine the fluctuations of the time-averaged entropy production around the nonequilibrium stationary state.

### 5.1. Entropy Production

The transition probabilities per unit time are

(83) $$W_{1}=V\;k_{1}^{+}\;a,\;W_{-1}=k_{1}^{-}\;N_{X},\;W_{2}=k_{2}^{+}\;N_{X},\;W_{-2}=V\;k_{2}^{-}\;b.$$

The corresponding rates per unit volume are

(84) $$\begin{array}{cccc} w_{1}\left(x\right) & =k_{1}^{+}\;a,\; & w_{-1}\left(x\right) & =k_{1}^{-}\;x,\\ w_{2}\left(x\right) & =k_{2}^{+}\;x,\; & w_{-2}\left(x\right) & =k_{2}^{-}\;b. \end{array}$$

Thus

(85) $$v_{1}=k_{1}^{+}\;a-k_{1}^{-}\;x,\;v_{2}=k_{2}^{+}\;x-k_{2}^{-}\;b,$$

and

(86) $$Q_{1}=k_{1}^{+}\;a+k_{1}^{-}\;x,\;Q_{2}=k_{2}^{+}\;x+k_{2}^{-}\;b.$$

The concentration obeys the chemical Langevin equation

(87) $$dx=\left(v_{1}-v_{2}\right)dt+\frac{1}{\sqrt{V}}\left(\sqrt{Q_{1}}\;dZ_{1}-\sqrt{Q_{2}}\;dZ_{2}\right),$$

with the Itô interpretation introduced in Section 2. Thermodynamic state functions are evaluated using the Stratonovich chain rule derived in Section 3. The affinities are

(88) $$\frac{A_{1}}{k_{B}\;T}=\ln\frac{k_{1}^{+}\;a}{k_{1}^{-}\;x},\;\frac{A_{2}}{k_{B}\;T}=\ln\frac{k_{2}^{+}\;x}{k_{2}^{-}\;b}.$$

For a Gillespie trajectory, the entropy production is calculated from Equations (30) and (33). In the ELE description, Equation (48) gives

(89) $$d_{i}s=\frac{A_{1}\;v_{1}+A_{2}\;v_{2}}{T}\;dt+\frac{1}{\sqrt{V}\;T}\left(A_{1}\;\sqrt{Q_{1}}\circ dZ_{1}+A_{2}\;\sqrt{Q_{2}}\circ dZ_{2}\right).$$

**Figure 1:**
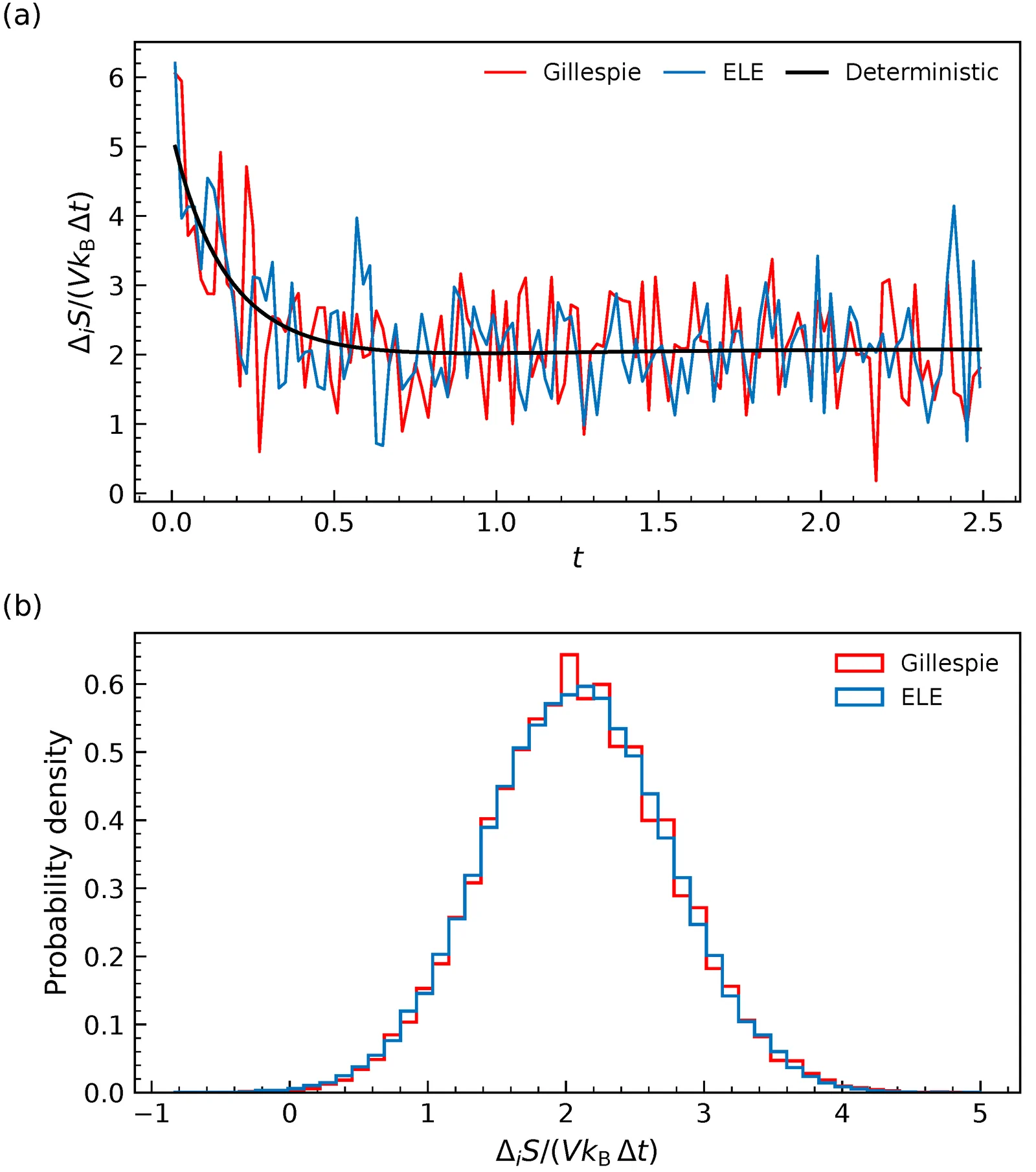
Entropy production for the open linear reaction process. (**a**) Entropy-production rate $\Delta_{i}S/\left(V\;k_{B}\;\Delta t\right)$ over successive intervals Δt along a Gillespie trajectory (red), an independent ELE trajectory (blue), and the deterministic evolution (black). (**b**) Stationary probability density of the same quantity for the Gillespie and ELE descriptions. The parameters are $k_{1}^{+}a=4$, $k_{1}^{-}=1$, $k_{2}^{+}=1$, and $k_{2}^{-}b=1$, with V=500 and Δt=0.02. In panel (**a**), x(0)=0.8. In panel (**b**), $x_{s}=2.5$ and $4\times 10^{4}$ independent intervals are sampled for each stochastic description, with initial states drawn from the corresponding stationary distribution.

**Figure 2:**
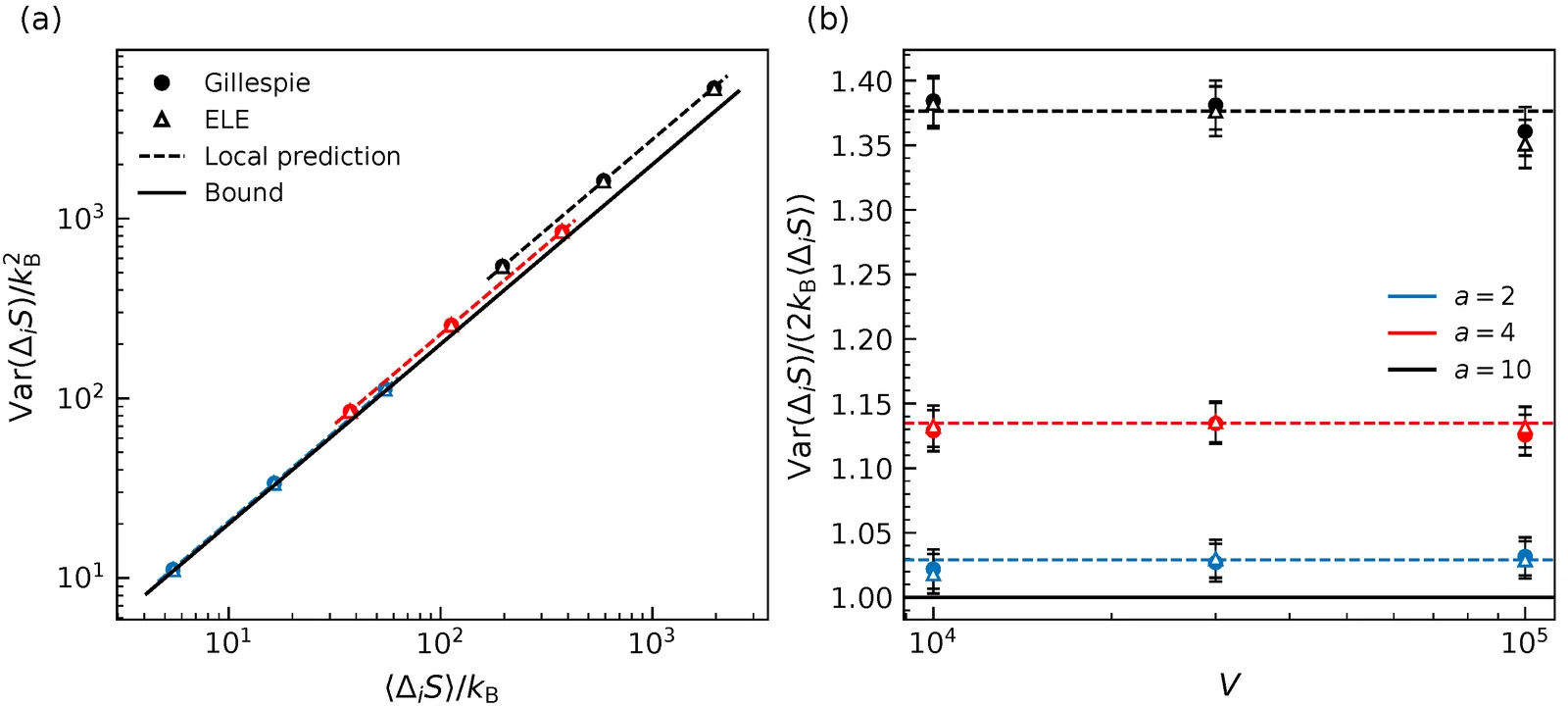
Numerical test of the entropy-production uncertainty relation for the open linear reaction process. (**a**) Variance of $\Delta_{i}S$ versus its mean for Gillespie simulations (filled circles) and ELE simulations (open triangles). Dashed lines are the predictions of Equations (90) and (91); the solid black line is $\mathrm{Var}\left(\Delta_{i}S\right)=2\;k_{B}\;\langle \Delta_{i}S\rangle$. (**b**) Ratio $\mathrm{Var}\left(\Delta_{i}S\right)/\left(2\;k_{B}\;\langle \Delta_{i}S\rangle \right)$ as a function of *V*. Dashed horizontal lines are the corresponding predictions and the solid black line marks the lower bound. The values $k_{1}^{+}a=2,4,10$ are shown, while $k_{1}^{-}=k_{2}^{+}=k_{2}^{-}b=1$. The initial concentration is x(0)=1.1, $\Delta t=10^{-3}$, and $V=10^{4}$, $3\times 10^{4}$, or $10^{5}$. Each point is obtained from $4\times 10^{4}$ independent realizations for each stochastic description and parameter set. Error bars in panel (**b**) are 95% intervals.

**Figure 3:**
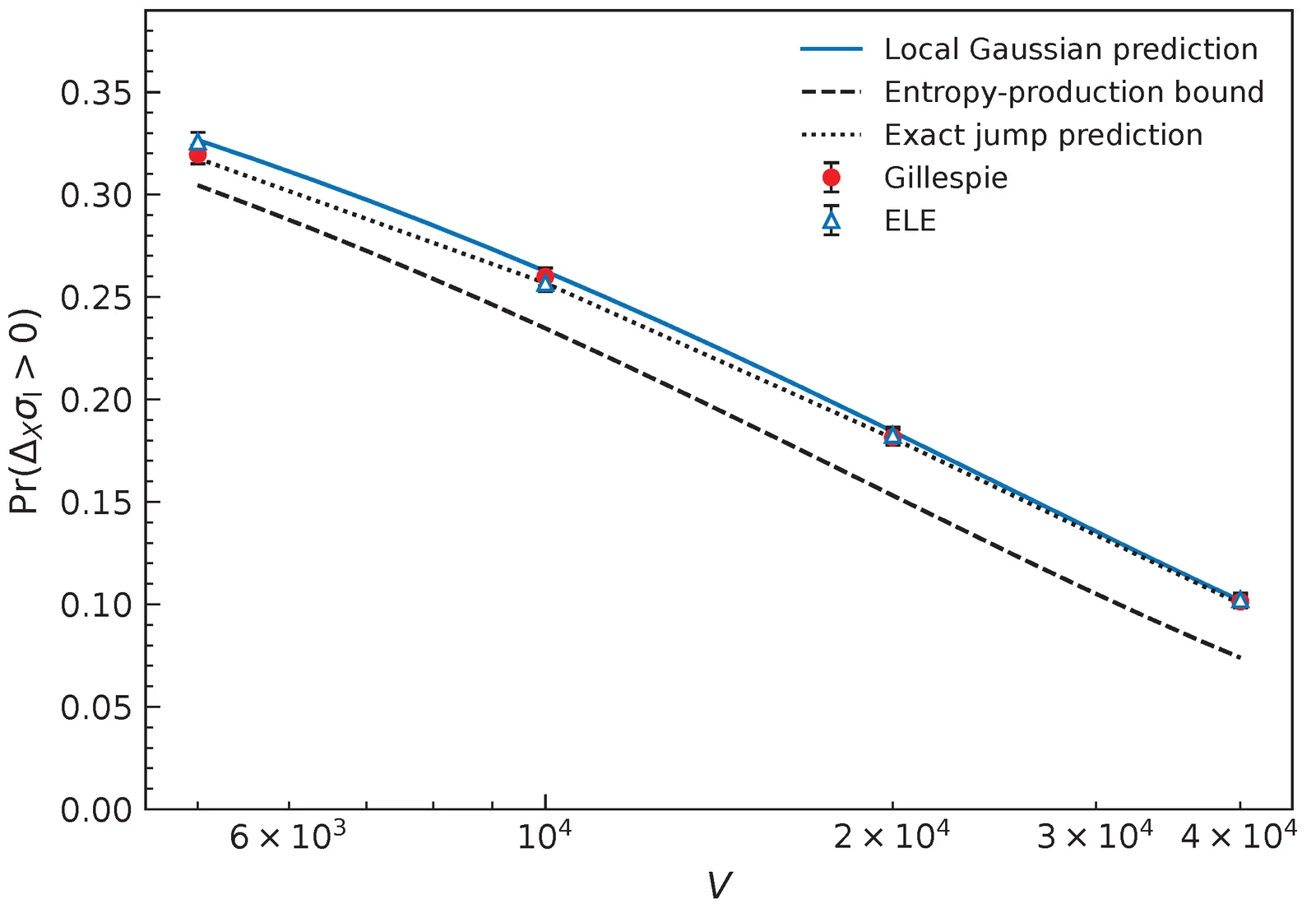
Probability of a reversal of the Glansdorff–Prigogine contribution for the open linear reaction process. Gillespie results (filled circles) and ELE results (open triangles) are compared with Equation (96), the bound (80), and the result obtained from the endpoint distribution of the linear jump process. The parameters are $k_{1}^{+}a=1.05$, $k_{1}^{-}=1$, $k_{2}^{+}=1$, and $k_{2}^{-}b=1$, with x(0)=0.98 and Δt=0.02. The volumes are $V=5\times 10^{3}$, $10^{4}$, $2\times 10^{4}$, and $4\times 10^{4}$, and each point is based on $4\times 10^{4}$ independent realizations for each stochastic description and volume. The condition is $\Delta_{X}\sigma_{I}>0$; error bars are 95% intervals.

The jump process was simulated with the Gillespie algorithm. For the ELE calculations, Equation (87) was written in its equivalent Stratonovich form and integrated with the stochastic Heun method. The $O(V^{-1})$ correction to the drift associated with this transformation was included in the numerical integration. The integration step was Δt/20 for Figure 1 and Δt/40 for Figure 2 and Figure 3. Entropy is expressed in units of $k_{B}$. No separate value of *T* is required, since the affinities enter the calculations through the ratios $A_{\rho }/\left(k_{B}T\right)$ in Equation (88).

Figure 1 compares the jump and ELE descriptions directly. The entropy-production rate is estimated from the entropy produced during successive intervals Δt. Panel (a) shows one Gillespie trajectory, one independent ELE trajectory and the deterministic result. Panel (b) compares the stationary distributions obtained from the two stochastic descriptions.

The comparison in Figure 1 concerns the first step of the present construction: the passage from the jump dynamics to the ELE equations. The agreement is not restricted to the deterministic evolution. The ELE description also reproduces the amplitude and the stationary distribution of the entropy-production fluctuations. This provides a numerical check of the weak-noise description derived in Section 3. The local relations between the mean and variance derived in Section 4 are tested separately below, using ensembles that start from the same prescribed composition.

For a fixed initial concentration *x*, Equations (59) and (60) give, for the entropy produced in the whole volume,

(90) $$\langle \Delta_{i}S\rangle =V\;\frac{A_{1}\;v_{1}+A_{2}\;v_{2}}{T}\;\Delta t,$$

and

(91) $$\mathrm{Var}\left(\Delta_{i}S\right)=\frac{V\;\Delta t}{T^{2}}\left(Q_{1}\;A_{1}^{2}+Q_{2}\;A_{2}^{2}\right).$$

Figure 2 compares these expressions with Gillespie and ELE simulations for different values of *V* and $k_{1}^{+}a$.

Figure 2 tests both moments entering the uncertainty relation. Two approximations enter the local predictions: the diffusion approximation at large volume and the evaluation of the coefficients at the initial composition over a short interval. Increasing *V* improves the former, but does not remove the error due to their evolution during a fixed Δt. The ratio $\mathrm{Var}\left(\Delta_{i}S\right)/\left(2\;k_{B}\;\langle \Delta_{i}S\rangle \right)$ remains larger than one over the range shown and is close to the value obtained from Equations (90) and (91). This supports the uncertainty relation in the regime where both approximations apply. The departure of the ratio from unity increases with $k_{1}^{+}a$, in agreement with the dependence on the affinities contained in Equation (61).

### 5.2. Glansdorff–Prigogine Contribution

The deterministic evolution of the concentration is

(92) $$\dot{x}=v_{1}-v_{2},$$

and therefore

(93) $$\frac{d}{dt}\left(\frac{A_{1}}{T}\right)=-\frac{k_{B}}{x}\;\dot{x},\;\frac{d}{dt}\left(\frac{A_{2}}{T}\right)=\frac{k_{B}}{x}\;\dot{x}.$$

Substitution into Equation (73) gives

(94) $$d_{X}\sigma_{I}=-\frac{k_{B}}{x}\;{\dot{x}}^{\;2}\;dt+\frac{k_{B}\;\dot{x}}{\sqrt{V}\;x}\left(-\sqrt{Q_{1}}\circ dZ_{1}+\sqrt{Q_{2}}\circ dZ_{2}\right).$$

The first term is the classical contribution. The second has no definite sign and can make the increment positive.

Its variance is

(95) $$\frac{\mathrm{Var}\left(\Delta_{X}\sigma_{I}\right)}{\Delta t}=\frac{k_{B}^{2}}{V\;x^{2}}\;{\dot{x}}^{\;2}\left(Q_{1}+Q_{2}\right),$$

and Equation (76) becomes

(96) $$\mathrm{Prob}\left(\Delta_{X}\sigma_{I}>0\right)=\frac{1}{2}\;\mathrm{erfc}\left[\sqrt{\frac{V\;\Delta t}{2}}\;\frac{|\dot{x}|}{\sqrt{Q_{1}+Q_{2}}}\right].$$

For this one-dimensional system, the integrated contribution can also be expressed in terms of the concentrations at the beginning and at the end of the interval. From

(97) $$d_{X}\sigma_{I}=-k_{B}\;\frac{\dot{x}}{x}\circ dx$$

and

(98) $$\dot{x}=k_{1}^{+}\;a+k_{2}^{-}\;b-\left(k_{1}^{-}+k_{2}^{+}\right)x,$$

one obtains

(99) $$\begin{array}{c} \Delta_{X}\sigma_{I}=k_{B}[\left(k_{1}^{-}+k_{2}^{+}\right)\left(x_{f}-x_{i}\right)\\ \;\;\;\;\;\;\;\;-\left(k_{1}^{+}\;a+k_{2}^{-}\;b\right)\ln\left(\frac{x_{f}}{x_{i}}\right)], \end{array}$$

where $x_{i}$ and $x_{f}$ are the concentrations at the beginning and at the end of the interval. For the ELE process, Equation (99) is equivalent to Equation (97). Since its right-hand side only depends on the two endpoint concentrations, it can also be evaluated along a Gillespie trajectory. This is the quantity used in Figure 3.

The endpoint distribution of the linear jump process can be calculated without a Gaussian approximation. Starting from $N_{i}=V\;x_{i}$ particles, each initial particle survives the interval with probability $p=\exp[-\left(k_{1}^{-}+k_{2}^{+}\right)\;\Delta t]$. The final population is the sum of two independent random variables: a binomial variable with parameters $N_{i}$ and *p*, and a Poisson variable with mean $V\;\left(k_{1}^{+}a+k_{2}^{-}b\right)\;\left(1-p\right)/\left(k_{1}^{-}+k_{2}^{+}\right)$. Summing this distribution over the endpoint populations for which Equation (99) is positive gives the jump-process benchmark in Figure 3.

Figure 3 compares the Gillespie and ELE simulations with the local Gaussian result in Equation (96). The exact endpoint benchmark accounts for both the discrete population changes and the evolution during the finite observation interval. In particular, a finite fraction of the jump-process intervals have no net population change. Such intervals are excluded by the strict condition $\Delta_{X}\sigma_{I}>0$, whereas they have zero probability in the continuous Gaussian description. Population discreteness therefore contributes to the difference from the local prediction, but agreement with the exact endpoint benchmark does not identify it as the only source of that difference. Increasing *V* does not by itself remove the error from freezing the coefficients over Δt.

The lower bound (80) is also satisfied throughout the range shown. It constrains the reversal probability through the leading local mean entropy production over the same interval. Its validity for these jump-process data is a numerical observation; the derivation establishes the bound within the local Gaussian approximation.

### 5.3. Fluctuations Around the Stationary State

We finally consider the entropy production averaged over times longer than the relaxation time. This is different from the short-time stationary distribution shown in Figure 1b. The stationary concentration, relaxation coefficient and stationary current are

(100) $$\begin{array}{ccc} x_{s}=\frac{k_{1}^{+}\;a+k_{2}^{-}\;b}{k_{1}^{-}+k_{2}^{+}},\; & L_{s}=-\left(k_{1}^{-}+k_{2}^{+}\right),\; & J_{s}=v_{1}\left(x_{s}\right)=v_{2}\left(x_{s}\right), \end{array}$$

and the stationary entropy-production rate is

(101) $$\sigma_{I,s}=\frac{J_{s}}{T}\left(A_{1,s}+A_{2,s}\right)=k_{B}\;J_{s}\ln\frac{k_{1}^{+}\;a\;k_{2}^{+}}{k_{1}^{-}\;k_{2}^{-}\;b}.$$

For an observation time *t*, we define

(102) $${\bar{\sigma }}_{t}=\frac{1}{t}\int_{0}^{t}d_{i}s.$$

Writing

(103) $$x=x_{s}+\frac{\eta }{\sqrt{V}},$$

the leading concentration fluctuation obeys

(104) $$d\eta =L_{s}\;\eta \;dt+\sqrt{Q_{1,s}}\;dZ_{1}-\sqrt{Q_{2,s}}\;dZ_{2}.$$

The concentration fluctuations and $\sigma_{\mathrm{II}}$ are driven by the same Wiener processes and are therefore correlated. Taking this correlation into account gives, for $t\gg \;|L_{s}{|}^{-1}$,

(105) $$\mathrm{Var}\;\left({\bar{\sigma }}_{t}\right)≃\frac{{\left[\left(A_{1,s}+A_{2,s}\right)/T\right]}^{2}}{V\;t\;{\left(k_{1}^{-}+k_{2}^{+}\right)}^{2}}\left[{\left(k_{2}^{+}\right)}^{2}Q_{1,s}+{\left(k_{1}^{-}\right)}^{2}Q_{2,s}\right].$$

The distribution of ${\bar{\sigma }}_{t}$ is Gaussian in this approximation, with a variance proportional to 1/(Vt). This recovers the behavior obtained previously from the ELE description around a nonequilibrium stationary state [29]. The present derivation also shows why the concentration fluctuations and the fluctuating contribution to entropy production cannot be treated independently: both are generated by the same reaction events.

## 6. Conclusions

In this work, we have derived the ELE evolution equations for thermodynamic state functions from a chemical Markov jump process. The changes of these functions are first expressed as finite differences associated with individual reaction events. In the weak-noise limit, their expansion over a mesoscopic time interval leads to the Stratonovich chain rule used in the ELE formulation. This prescription applies to the thermodynamic state functions; the concentration dynamics itself is described by the Itô chemical Langevin equation.

The entropy production separates into the systematic contribution $\sigma_{I}$ and the contribution $\sigma_{\mathrm{II}}$ associated with the fluctuating reaction rates. The former remains non-negative at every composition, while the latter has no definite sign. The mean and variance of the total entropy produced over a mesoscopic time interval obey a bound of the thermodynamic uncertainty form. Here, this relation follows directly from the forward and reverse reaction rates and applies locally in composition, without requiring a stationary state.

We have also considered the Glansdorff–Prigogine contribution associated with changes of the thermodynamic forces. The classical nonpositive term is supplemented, at finite volume, by a fluctuating contribution with no definite sign. Its variance can be calculated from the same quantities that determine the entropy-production fluctuations. Within the local Gaussian approximation, this gives the probability of observing an increment with the opposite sign to the classical contribution. A lower bound on this probability follows from the entropy produced during the same time interval. Within this approximation, small entropy production limits how reliably the classical sign can be observed. Greater dissipation relaxes this restriction, but is not sufficient to guarantee reliable agreement with the classical evolution.

The linear reaction scheme provides a direct comparison with Gillespie simulations for entropy-production fluctuations and GP reversals. The comparison separates the diffusion approximation from the additional approximation of fixed coefficients over the observation interval. The exact endpoint distribution provides a benchmark for the GP observable that includes both finite-time evolution and population discreteness. The same formulation also recovers the long-time entropy-production fluctuations obtained previously within the ELE approach.

Two questions follow directly from these results. The first concerns the reliability of the GP sign near stationary compositions and bifurcations, where the leading local calculation may no longer suffice. The finite changes along jump trajectories provide a starting point for retaining the terms and non-Gaussian fluctuations needed in these regimes. The second concerns which thermodynamic predictions survive the elimination of reaction intermediates or channels. Even the present linear example has a stationary throughput that cannot be inferred from the mean change of its internal population alone. Comparing the full and reduced descriptions would identify what additional reaction information must be retained. Extensions to nonideal mixtures and spatially distributed systems are also possible directions. For nonideal mixtures, the rates must remain consistent with the chemical potentials, and the GP sign requires convexity on the accessible composition variations.

## Data Availability

No external data sets were used in this study. All numerical data shown in Figure 1, Figure 2 and Figure 3 are generated by the three Jupyter notebooks provided as Supplementary Materials; the notebooks contain the parameters and fixed random-number seeds required to reproduce the calculations.

## Supplementary Materials

The following supporting information can be downloaded at: https://www.mdpi.com/article/10.3390/e28101154/s1. The numerical calculations are provided as three self-contained Jupyter notebooks: Notebook S1 generates Figure 1, Notebook S2 generates Figure 2, and Notebook S3 generates Figure 3. Each notebook generates the numerical data and the corresponding PDF/EPS figure files. Simulation parameters and random-number seeds are specified in the notebooks.
